# Gait Disorders Questionnaire–Promising Tool for Virtual Reality Designing in Patients With Parkinson's Disease

**DOI:** 10.3389/fneur.2019.01024

**Published:** 2019-09-24

**Authors:** Zuzana Kosutzka, Alice Kusnirova, Michal Hajduk, Igor Straka, Michal Minar, Peter Valkovic

**Affiliations:** ^1^2nd Department of Neurology, Faculty of Medicine, Comenius University, Bratislava, Slovakia; ^2^Department of Psychology, Faculty of Arts, Comenius University, Bratislava, Slovakia; ^3^Department of Psychiatry, Faculty of Medicine, Comenius University, Bratislava, Slovakia; ^4^Centre of Experimental Medicine, Institute of Normal and Pathological Physiology, Slovak Academy of Sciences, Bratislava, Slovakia

**Keywords:** Parkinson's disease, gait, freezing of gait, virtual reality, neurorehabilitation

## Abstract

**Background:** Gait disorders (GD) are frequent and disabling symptoms in patients with Parkinson's disease, mostly because they significantly limit mobility and often lead to fear of falls or actual falls. Nowadays, rehabilitation is considered to be the most effective nonpharmacological approach to reduce risk of falls. Using paradigms in virtual reality (VR) is a promising tool in neurorehabilitation because of the potential improvement in motor learning and improvement in daily functioning by replicating everyday real-life scenarios.

**Objective:** To identify the most prevalent everyday situations which impair gait in PD that could be simulated in virtual reality (VR) environment.

**Methods:** A newly developed self-report questionnaire consisting of 15 binary response items (YES/NO) encompassing everyday walking situations was administered to 62 patients diagnosed with idiopathic PD according to MDS Clinical Diagnostic Criteria. We included patients able to walk unassisted for at least 10 min and without significant cognitive impairment. Mokken Scale Analysis was used to evaluate psychometric properties of the scale.

**Results:** Questionnaires from 58 patients were analyzed (31 men, age = 63 ± 9.9 y, disease duration = 7.02 ± 4.03 y, LEDD = 1115 ± 549.4 mg, H&Y = 2.4 ± 0.6). Only 10 items (out of 15) were identified as scalable and these were included in Gait Disorders Questionnaire (GDQ). The most prevalent trigger of gait disorders was walking under time pressure, followed by gait in crowded places and walking while dual-tasking. The total score of GDQ significantly correlated with the disease duration (*r*_s_ = 0.347, *p* = 0.008) and modified H&Y staging (*r*_s_ = 0.288, *p* = 0.028).

**Conclusion:** With the use of GDQ we identified the most prevalent everyday transition activities that provoke gait disorders in patients with PD. The results may be useful for further development and systematic application of VR paradigms for physiotherapy of PD patients.

## Introduction

Gait disorders (GD) are highly prevalent and incapacitating symptoms among patients with Parkinson's Disease (PD), which often lead to falls, with immobilization, impaired quality of life, and reduced life expectancy (1). So far, postural instability and related GD have been considered to be one of the most enigmatic symptoms in PD. In comparison with tremor, bradykinesia and rigidity, GD are, by far, the least understood. The clinical picture of GD may vary from subtle subclinical gait asymmetries to the complete blockage of gait initiation with ineffective stepping (2). Typical gait in PD includes stooped posture, semi-flexed upper and lower limbs, and shuffling steps. Asymmetrically reduced arm swing is also a characteristic feature and is often the only symptom for years within the initial stages of the disease (3). GD in PD include constantly present signs or episodic phenomena (e.g., freezing of gait—FoG, festinations, hesitations) (4).

Freezing of gait (FoG) is a severely debilitating GD which affects around 60% of the patients predominantly in advanced stages of the disease (5). FoG is defined as a brief episode during which it is impossible to execute a step and patients often report a feeling of “feet glued to the floor” (6). Despite the immense research efforts, the pathophysiology of FoG is not entirely understood. Even less explored GD typical for PD patients is festination of gait (FEG), characterized by a propensity to lean forward when walking (7). Typically, the patient involuntarily moves ahead with short, accelerating steps, often on tiptoe, with the trunk flexed forward, and the legs bend stiffly at the hips and knees.

Several questionnaires and scales with or without additional accessories are available for the assessment of GD in PD; for review see publication by Bloem et al. (8). In clinical practice gait is assessed according to Movement Disorders Society—Unified PD Rating Scale (MDS-UPDRS) with patient as well as clinician rated section including gait, balance and FoG (9). The severity of FoG can be further assessed from patient's point of view using Freezing of Gait Questionnaire (10). Other patient reported questionnaires are available but either they are not sufficiently focusing on gait (11) or they are not addressing disease specific gait issues (12, 13).

Patients often report GD triggering situations in everyday life, but due to the unpredictable nature, it is not easy to trigger them in clinical and research settings. Clinical scales include various provoking strategies to challenge gait and reveal subclinical GD including time measured gait, clockwise and counter-clockwise turns, walking over the obstacles, etc. (14, 15). In the case of FoG the dual tasking (e.g., talking while walking), gait in tight quarters, and turns can be used (16). To our knowledge, no provoking tricks of FEG have been thus far identified.

The pharmacological treatment of PD gait disorders is rather limited due to the complexity of the pathophysiological background. New hope was brought into the field with new targets of deep brain stimulation (pedunculopontine nucleus, substantia nigra pars reticulata) but the results are at this time, not entirely conclusive (17, 18). Due to the lack of satisfactory treatment options the effect of exercise progressively comes to the fore. At this time, the most efficient non-pharmacological treatment of gait disorders is physical therapy with a sustained effect in the long term (19). The main principle includes the constant switch of attention toward the movements with desired amplitude and pace with or without the use of various external cues (20). In this framework, including virtual reality (VR) component to regular gait training showed added value with the improvement of physical performance and gait during challenging situations (21, 22).

VR paradigms are potentially an efficient tool to simulate more natural everyday situations. VR is defined as electronic simulations of environments experienced via head mounted eye goggles and wired clothing enabling the end user to interact in realistic, three-dimensional scenarios. VR ranges from non-immersive to fully immersive, depending on the degree to which the user is isolated from the physical surroundings when interacting with the virtual environment (23). Non-immersive VR has been repeatedly used in physiotherapy of neurological diseases including PD (24), but have been used to a lesser extent in GD research (25). Several studies have confirmed the utility of VR paradigms, especially in the research of FoG in PD in combination with fMRI (26, 27). Investigation of Maidan et al. about the role of prefrontal cortex in falls in PD showed that combined motor-cognitive training intervention, which includes VR paradigms, may result in changes to the prefrontal activation pattern and it improves functional abilities, reduces falls, and risk of fall (28). VR combined with treadmill training promotes the development of motor and cognitive strategies for obstacle navigation which may be transferred to everyday situations (21). However, there are no standardized VR environments that are used for research and physiotherapy of GD in PD.

A questionnaire that encompasses the everyday activities provoking the GD in PD is lacking. Therefore, the main aim of the current study was to develop a quick and patient-friendly tool which would identify the everyday situations impairing the gait in PD patients. Taking into account the fact that physiotherapy combined with VR paradigms seems to be an efficient and safe therapy option for GD in PD we adjusted the designing of the tool with a future potential to simulate these situations in the VR environment.

## Methods

### Participants

All subjects were recruited consecutively during a 3-month period from the Movement Disorder Outpatients Clinic of the 2nd Department of Neurology, University Hospital Bratislava. The diagnosis of PD was established according to the MDS Clinical Diagnostic Criteria (29) and the disease duration was set to a minimum of 1 year. Only patients with modified Hoehn and Yahr stages (H&Y) 1–4 (9, 30) were included and they had to be able to walk unassisted at least 10 min. All participants had to be on a stable dopaminergic treatment for a minimum of the last 3 months. Levodopa equivalent daily doses (LEDD) were calculated according to standard reporting (31). The protocol was completed during the best “on” state after taking their usual morning antiparkinsonian medication. Only patients who were able to understand and cooperate with study procedures were recruited. The cognitive status was established through a comprehensive clinical interview including assessment of daily functioning and clinical examination conducted by a movement disorder specialist. Patients had to be able to complete the questionnaire on their own. Subjects with diagnosed dementia were excluded from the study. Patients with other comorbidities (ophthalmological, auditory, orthopedic and musculoskeletal diseases) that could interfere with the ratings were also excluded.

### Protocol

Firstly, during semi-structured comprehensive interview demographic information was collected and the cognitive status was screened. The evaluation also included a question “Do you feel that your feet get glued to the floor?” and patients with positive answer were classified as “probable freezers” as suggested by Snijders et al. (32). The interview was followed by a neurological examination with special focus on PD. Secondly, patients were instructed and asked to fill in a questionnaire measuring their perception of gait across 15 everyday situations that include walking. All questions had a binary response format with answers “YES” or “NO,” if the situation mentioned is or is NOT triggering the subjective feeling of gait disorders (see Supplementary Figure 1). The selection of each item was based on previous literature review (33–36) and our clinical experience. The study was approved by the local Ethics Committee (Academic Derer's University Hospital, Bratislava) and all patients provided written informed consent in accordance with the Declaration of Helsinki.

### Statistical Analysis

Descriptive statistical analysis was performed to assess demographic and clinical data. To compare differences between groups (patients with and without postural instability), we applied Mann-Whitney *U*-test due to a violation of assumption of normality. Analysis of covariance was used to analyse the possible differences in questionnaire scores accounting for age and gender. Spearman's rank correlation coefficient (rho, *r*_s_) was used to evaluate the strength of the association between the Gait Disorders Questionnaire (GDQ) and clinical variables. *P*-values ≤ 0.05 were considered to be significant. The reliability of the questionnaire was assessed with Cronbach's alpha. For all other analyses IBM SPSS version 23 was used. With the aim of identification of homogenous unidimensional set of items, Mokken Scale Analysis was used to assess the psychometric properties of the questionnaire and was performed with Mokken package in R (37). The minimally acceptable scaling coefficient was set to 0.30 (Item *H*_j_). The higher value of *H*_j_ indicates better discriminatory power of the item.

#### Mokken Analysis at a Glance

Mokken Scale Analysis (MoSA) is model of measurement based on the Guttman scaling, using the probabilistic approach and is considered as the nonparametric version of Item Response Theory (38, 39). Several assumptions for MoSA were proposed. The first assumption is that items are hierarchically ordered based on their difficulties. In dichotomous items difficulty represents the percentage of affirmative/correct answers. Another MoSA assumption is unidimensionality which means that all items of scale are measuring the same latent construct (trait). Assumption of monotonically non-decreasing item response functions (e.g., monotonicity) means that higher item scores are expected on higher degree of latent trait. These assumptions are necessary for using Monotone Homogeneity Model. It is also possible to fit a more restrictive model which is called Double Monotonicity Model. In this model, an additional assumption about non-intersection of item characteristics curves is proposed.

The last general assumption is typical for various statistical models and it means that response of the individual to the item is not influenced by response on the other items within the scale or test (40).

Psychometric properties scale in MoSA is evaluated using scalability coefficients (*H*_j_) (41). Coefficients range from 0 to 1 and they represent an accuracy of scale order respondents based on their raw score (42). These coefficients are used for evaluation of the homogeneity of a set of items. Items with higher *H*_j_ have better discrimination power (ability to order respondents). Scales with mean *H*_j_ <0.30 are not considered unidimensional. Scales with *H*_j_ 0.30 ≤ *H*_j_ ≤ 0.40 are considered as weak scales. Medium strength scales have 0.40 ≤ *H*_j_ ≤ 0.50. When the mean *H*_j_ is >0.50, the scale is considered strong. The minimally acceptable value of the scaling coefficient for items is 0.30 (Item *H*_j_) (38).

## Results

### Patient Characteristics

We gathered 62 fully completed questionnaires. Four patients did not report any problems with gait and so they were excluded from further analyses. The final analysis included questionnaires from 58 patients (31 men, 27 female). The mean age was 63 ± 9.9 y with mean disease duration of 7.02 ± 4.03 y, mean LEDD 1,115 ± 549.4 mg, and mean H&Y stage 2.4 ± 0.6.

Based on the positive answer to the question “Do you feel that your feet get glued to the floor while walking, making a turn or when trying to initiate walking (freezing)?” 41 patients (70.7%) were classified as “probable freezers.” With regard to H&Y staging, 29 patients (50%) were considered to have postural instability (H&Y ≥ 2.5).

### Psychometric Properties of Gait Disorders Questionnaire (GDQ)

**Figure 1:**
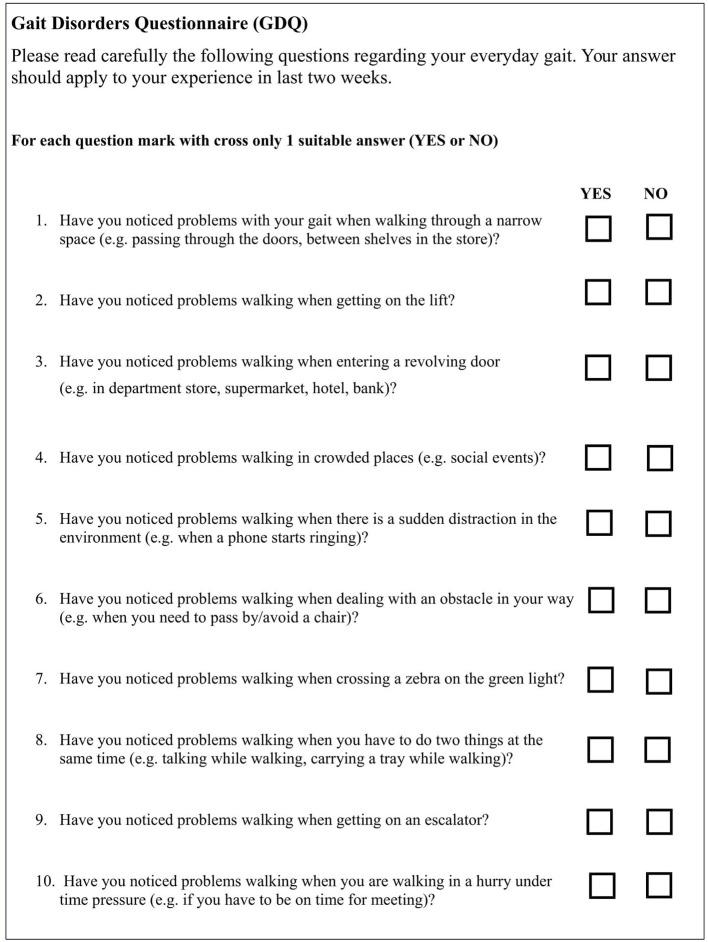
The gait disorders questionnaire.

The satisfactory psychometric properties were met in 10 out of 15 items (*H*_i_ >0.30 and Item total correlation higher than 0.300). These ten items were included in GDQ, see Figure 1. The Cronbach alpha of GDQ was α = 0.852 which confirms a high internal consistency and reliability of the scale. List of items with corresponding psychometric features is shown in Table 1. All items had sufficient *H*_i_ coefficient and item total correlation (ITC) values were higher than 0.30. The scaling coefficient (Ht) for all patients was 0.502 (SE = 0.066). Molenaar-Sijtsma reliability index was 0.859 which also confirms a high reliability with regard to MoSA. Results from monotonicity and non-intersection assessment showed adequate properties for almost all items (Supplementary Table 1). Item “Narrow spaces” had some violation of monotonicity and non-intersections. The item was retained in the analysis due to its known, high prevalence in patients with FoG and good item-total correlation. MoSA in overall support sound psychometric properties of the GDQ.

**Table 1:** Ranking of the gait impairment situations in all patients (descending order from the most prevalent–“Time stress” to the least prevalent–“Zebra crossing”) with corresponding H_i_ coefficients.

	**Mean**	**STD**	**%**	**H_**i**_**	**SE**	**ITC**
Time stress	0.724	0.45	72.1	0.441	(0.14)	0.359
Crowded places	0.586	0.49	58.6	0.518	(0.07)	0.568
Dual-tasking	0.534	0.50	53.4	0.390	(0.10)	0.451
Sudden change of situation	0.500	0.43	50.0	0.490	(0.08)	0.597
Narrow spaces	0.500	0.50	50.0	0.310	(0.10)	0.364
Obstacle on the way	0.466	0.50	46.6	0.517	(0.08)	0.630
Getting on escalator	0.379	0.49	37.9	0.464	(0.10)	0.537
Revolving doors	0.379	0.50	37.9	0.582	(0.09)	0.689
Getting on lift	0.328	0.47	32.8	0.655	(0.09)	0.723
Zebra crossing	0.241	0.50	24.1	0.736	(0.10)	0.670

*STD, standard deviation of mean; %, percentage of positive (“YES”) responses; H_j_, scalability coefficient; SE, standard error of H_j;_ ITC, item total correlation*.

### GDQ Scores and Correlations With Clinical Variables

**Figure 2:**
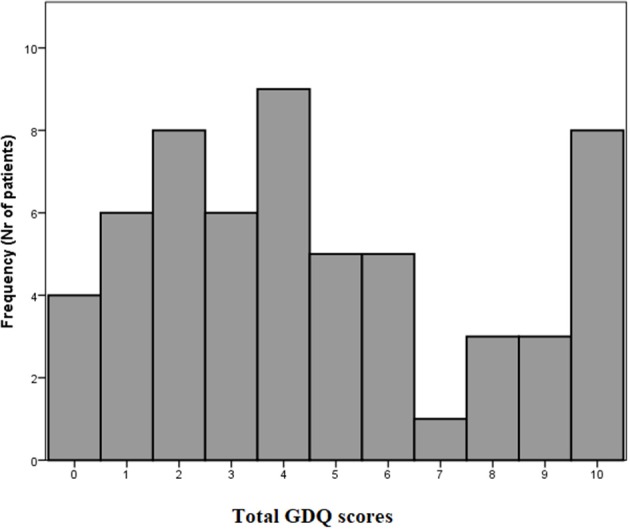
Frequency of gait disorders questionnaire (GDQ) scores in all patients.

The total GDQ score was considered as the sum of items to which a given participant responded “YES.” The mean GDQ score in our cohort was 4.6 ± 3.2 (see frequencies of total GDQ score ranking in Figure 2). The most important trigger that caused problems with gait in 72.1% of patients was walking under time pressure. This was followed by the gait in crowded place which was reported 58.6%. The third most prevalent trigger of GD reported by 53.4% was gait while dual-tasking. The individual ranking of all situations in GDQ is presented in Table 1. The total score of GDQ significantly correlated with the disease duration (*r*_s_ = 0.347, *p* = 0.008) and H&Y staging (*r*_s_ = 0.288, *p* = 0.028). There were no significant correlations of total GDQ with age (*r*_s_ = 0.009, *p* = 0.947) or LEDD (*r*_s_ = 0.020, *p* = 0.903).

### Gait Disorder Provoking Situations With Regard to Postural Impairment Based on Modified Hoehn and Yahr Staging

**Figure 3:**
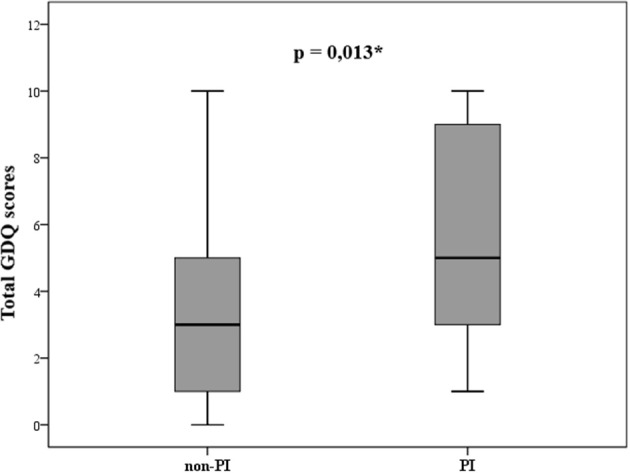
Statistically different gait disorders questionnaire (GDQ) scores in patients without (non-PI) and with (PI) postural instability according to modified Hoehn and Yahr staging (^*^p ≤ 0.05).

To evaluate potential differences in situations that may provoke postural instability, patients were divided into two groups; patients without postural instability (non-PI) who had H&Y score ≤ 2 (*n* = 29; “probable freezers” in non-PI group *n* = 15) and patients with postural instability (PI) and H&Y score ≥ 2.5 (*n* = 29; “probable freezers” in PI group *n* = 26). The total GDQ was significantly higher in patients with postural instability (*U* = 261, *p* = 0.013, mean GDQ score non-PI = 3.6 ± 2.9, PI = 5.7 ± 3.1, Figure 3). We found these significant group differences in GDQ scores also after accounting for gender and age [*F*_(1, 53)_ = 6.129, *p* = 0.017, eta2 = 0.104]. The patients with postural instability had significantly longer disease duration (*U* = 269.5, *p* = 0.018, mean non-PI = 5.9 ± 4.1, mean PI = 7.9 ± 4.5). There was no statistical difference in age (*U* = 359, *p* = 0.338) or LEDD (*U* = 209, *p* = 1.0) between groups. Table 2 shows the ranking of most prevalent situations in these two groups based on the mean prevalence for each situation.

**Table 2:** Prevalence of the most prevalent gait disorders provoking situations in GDQ (descending order from the most prevalent to the least prevalent).

**Without balance impairment (non-PI**, ***n*** **=** **29)**			**With balance impairment (PI**, ***n*** **=** **29)**		
**Situation**	**Mean**	**STD**	**Situation**	**Mean**	**STD**
1. Time stress	0.621	0.49	1. Time stress	0.828	0.38
2. Dual-tasking	0.552	0.48	2. Narrow spaces	0.690	0.50
3. Crowded places	0.517	0.50	3. Crowded places	0.655	0.48
4. Getting on escalator	0.345	0.30	4. Sudden change of situation	0.655	0.49
5. Sudden change of situation	0.345	0.45	5. Obstacle on the way	0.655	0.51
6. Narrow spaces	0.310	0.51	6. Dual-tasking	0.517	0.48
7. Revolving doors	0.310	0.48	7. Getting on lift	0.448	0.50
8. Obstacle on the way	0.276	0.47	8. Revolving doors	0.448	0.48
9. Getting on lift	0.207	0.41	9. Getting on escalator	0.414	0.50
10. Zebra crossing	0.103	0.47	10. Zebra crossing	0.379	0.47

*Non-PI, patients without postural instability; PI, patients with postural instability*.

## Discussion

The aim of the current study was to identify the most troublesome everyday situations that impair gait in patients with PD, which can be potentially used in the design of VR paradigms. We developed a10-item, self-report questionnaire of gait disorders with good psychometric properties, which can be used for quick and efficient assessment of gait impairing situations of patients with PD.

### The Most Prevalent Situations Provoking Gait Impairment

Our results show that the most prevalent trigger of gait impairment is walking under time pressure, which was indicated by 72% of the patients in our sample. Stress is undoubtedly connected with changes in motor performance. Animal studies with stress-inducing behavioral tests shown that walking under stress increases the base of support and reducing the stride length (43). Interestingly, increased cadence is typical for patients with balance impairment and oftentimes precedes the FoG episodes (44). Another stress-related response, that is highly prevalent in patients with PD, is anxiety. It may also be responsible for impaired gait performance (45). Anxiety can be intrinsic to the disease (46), but it can also develop specifically in relation to a physical symptom, such as FoG. In fact, longitudinal study confirmed that anxiety levels as measured by self-reported questionnaires are a strong predictor of future onset of FoG (47). The utility of VR paradigms using gait on a plank above a high pit to increase anxiety and trigger FoG was also confirmed previously (48).

The second most prevalent provoking situation of GD was walking in crowded places, which was reported in 58.6% patients. Walking in crowded places includes several factors which can impair gait in PD. Firstly, patients find it stressful to socialize with people (especially healthy) because they feel judged and perceived as physically disabled because of their appearance, which is, naturally, highly anxiety-provoking (49). Secondly, patients with PD and GD show impaired processing of sensory information (50). Therefore, crowded places are usually confined and can be perceived as a conflicting overload of visual information, so patients are prone to experience the sensory “jam.” This is also supported by the fact that gait in narrow spaces (e.g., doorways) is especially efficient in triggering FoG (34).

Gait impairment provoked by dual-tasking was the third most prevalent symptom, which was endorsed by 53.6% of the patients. Dual- and multi-tasking paradigms involve the execution of two and more simultaneous tasks, which are especially challenging for cognitive functions. These paradigms rely upon executive functions and the ability to divide attention between gait and a secondary task (51). Tessitore et al. (52) also reported that patients with FoG have impaired functional connectivity within the frontoparietal networks subserving attention functions. An additional study has shown that the challenge of dual-tasking is associated with higher gait variability and irregularities in gait rhythmicity (53). Dual-tasking can therefore unmask the subclinical gait disorders and it should be implemented in examination of Parkinsonian gait using VR paradigms.

The validity of the questionnaire was partially supported by association of GDQ scores and the disease duration. It is well known that the prevalence of gait and balance impairment is higher as the disease progresses (54), even though the highest prevalence of falls is in H&Y stage 3, when patients are able to walk relatively independently (55). Another interesting outcome of our study is the statistical difference of GDQ scores in patients with and without postural instability based on modified H&Y stage. It points to the potential to utilize the questionnaire in the identification of patients with postural instability and stratification of fall risk. Nevertheless, the cut-off scores, sensitivity and specificity of the questionnaire with regard to postural instability should be confirmed in a larger population sample.

### Designing the Virtual Reality Paradigms for Physiotherapy of Gait Disorders–Another Road to Personalized Medicine

**Figure 4:**
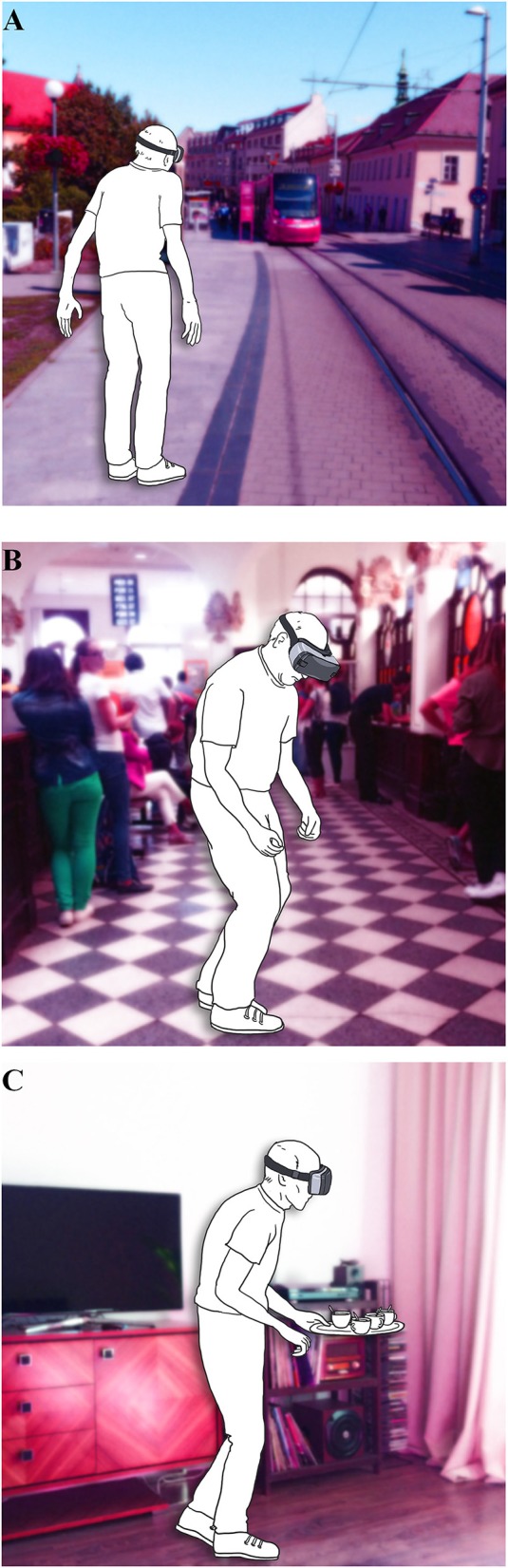
Illustrative examples of the VR paradigms based on the prevalence according to Gait Disorders Questionnaire **(A)** gait under time stress; **(B)** gait in crowded places; **(C)** gait under dual-tasking.

There is a growing body of evidence that a VR environment offers a new accessible approach to gait and balance research, for both, diagnosis and therapy (56). An important step for further development is the choice of VR tasks that would provide the most valuable and clinically relevant information. The examples of the three most prevalent situations derived from the results of our study simulated in VR environment is shown in Figure 4. Our questionnaire with concrete GD provoking situations unique to PD could mediate a more standardized approach to this method. Moreover, this could possibly enable the inclusion of larger patient population, and thus more rapidly advance the field of VR in gait and balance research of PD patients.

GD in PD are complex with regard to the pathophysiological background and clinical picture with a high need of a personalized approach to each patient (57). We suggest that the GDQ could be used in tailoring the gait and balance physiotherapy based on individual needs. The patient can indicate in the questionnaire the most troublesome gait impairment situations which can be easily chosen and simulated from 10-item VR battery. During the exposure to challenging VR tasks they can be instructed about coping and preventive strategies. Probably one of the most important outcomes of personalized physiotherapy is the efficient reduction of the fear of falling, as well as the actual incidence of falls. This is in congruence with the recent multi-center, randomized clinical trial in elderly adults including PD patients which showed a significant reduction in the fall rates when a non-immersive VR component was added to treadmill training as compared to treadmill walking alone (3).

### Limitations of the Study

Results of the current study need to interpreted in the light of several limitations. In this study, we included a subjective self-reporting questionnaire and therefore it is possible that patients were not reporting the situations that are troublesome objectively. We did not include the neuropsychological assessment which could impact the results. With regard to our patient population (relatively older patients with longer disease duration) we could have missed patients who had more sever cognitive deficits. These patients are also at higher risk of having GD (58), therefore, the prevalence and selection of the responses could be influenced. Our patient population with relatively longer disease duration could very likely experience motor fluctuations and dyskinesias which are known for great impact on gait. Motor complications of PD could therefore influence the outcomes of the study. In the objective clinical assessment, postural instability was only assessed using the pull test, but this was done by the same rater (A.K.) and it is still considered as a gold standard in the assessment of postural reactions (59). We did not include other clinical assessment of motor symptoms and gait that could lead to faulty classification of patients with and without postural instability and thus inaccurate H&Y staging. Nevertheless, the validity of the questionnaire was partly verified by the association with H&Y staging. Freezers were classified solely based on subjective personal experience without exact verification by a movement disorder specialist. Therefore, our results could be influenced by high prevalence of freezers in our cohort (70%), even though, the proportion of freezers in our study is consistent with previous research of this type (36).

## Conclusion

The Gait Disorders Questionnaire is a one-dimensional self-report measure with sound psychometric properties. Association with relevant clinical variables supports the validity of this newly developed scale although this has to be verified in larger patient samples using objective clinical assessment of gait.

We believe that examining patients in the VR environment is an effective way to trigger episodic and subclinical gait phenomenon in clinical setting. Additionally, the GDQ can be potentially used as a screening tool in order to apply suitable and personalized VR paradigms in the research, diagnosis and neurorehabilitation of GD in PD. In conclusion, more large-scale studies are needed to confirm the efficacy of VR technologies in PD and choosing the appropriate VR paradigms is a key step in advancing this promising field.

## Data Availability Statement

The datasets generated for this study are available on request to the corresponding author.

## Ethics Statement

This study was carried out in accordance with the recommendations of local Ethics Committee (Academic Derer's University Hospital, Bratislava) with written informed consent from all participants. All subjects provided written informed consent in accordance with the Declaration of Helsinki.

## Author Contributions

ZK and AK designed the research, drafted the manuscript, and with the cooperation of IS interpreted the results. MH processed the statistical analysis of data. MM and PV provided critical revisions of the manuscript. All authors have approved the final version of the manuscript.

### Conflict of Interest

The authors declare that the research was conducted in the absence of any commercial or financial relationships that could be construed as a potential conflict of interest.
