# Supplementary material for: Gait Disorders Questionnaire–Promising Tool for Virtual Reality Designing in Patients With Parkinson's Disease
Source: Front Neurol. 2019 Sep 24;10:1024. doi: 10.3389/fneur.2019.01024 (PMC6768968; doi:10.3389/fneur.2019.01024)
Supplement: Supplementary file 1 [file Image_1.pdf]

Supplementary Figure 1. Full 15-item questionnaire (items in bold were excluded after Mokken Scale Analysis and these items were omitted in Gait Impairment Questionnaire)

1. Have you noticed problems with gait when walking through a narrow space (e.g. passing through the doors, between shelves in the store)? ☐ ☐
2. Have you noticed problems walking when getting on the lift? ☐ ☐
3. Have you noticed problems walking when entering a revolving door (e.g. in department store, supermarket, hotel, bank)? ☐ ☐
4. Have you noticed problems walking in crowded places (e.g. social events)? ☐ ☐
5. Have you noticed problems walking when there is a sudden distraction in the environment (e.g. when a phone starts ringing)? ☐ ☐
6. Have you noticed problems walking when dealing with an obstacle in your way (e.g. when you need to pass by/avoid a chair)? ☐ ☐
7. Have you noticed problems walking when crossing a zebra on the green light? ☐ ☐
8. Have you noticed problems walking when you have to do two things at the same time (e.g. talking while walking, carrying a tray while walking)? ☐ ☐
9. **Have you noticed problems walking when there is a change in a surface (e.g. from a firm path to grass)?** ☐ ☐
10. **Have you noticed problems with gait when walking up the stairs?** ☐ ☐
11. **Have you noticed problems with gait when walking in dark environment (e.g. in the evening)?** ☐ ☐
12. Have you noticed problems walking when getting on an escalator? ☐ ☐

13. Have you noticed problems with gait when you are walking in a hurry under time pressure (e.g. if you have to be on time for meeting)? ☐ ☐

14. **Have you noticed problems with gait when walking on a soft terrain?** ☐ ☐

15. **Have you noticed problems with gait when walking in shoes in comparison with walking barefoot?** ☐ ☐
