# Supplementary material for: Gait Disorders Questionnaire–Promising Tool for Virtual Reality Designing in Patients With Parkinson's Disease
Source: Front Neurol. 2019 Sep 24;10:1024. doi: 10.3389/fneur.2019.01024 (PMC6768968; doi:10.3389/fneur.2019.01024)
Supplement: Supplementary file 2 [file Table_1.docx]

**Supplementary Table 1.** Summary of monotonicty values and non-intersection check of 10 items included in Gait Disorders Questionnaire (Monotonicity - assumption of monotonically non-decreasing item response functions, Non intersection - assumption that item characteristics curves do not intersect) ac, the number of active pairs; vi, the number of violations; zsig, the number of significant violations; crit, critical violation.

|  | Monotonicity check | | | | Non - intersection check P - matrix | | | |
| --- | --- | --- | --- | --- | --- | --- | --- | --- |
|  | #ac | #vi | #zsig | crit | #ac | #vi | #zsig | crit |
| **Narrow spaces** | 6 | 2 | 0 | 83 | 72 | 17 | 1 | 77 |
| **Getting on lift** | 1 | 0 | 0 | 0 | 72 | 4 | 0 | 0 |
| **Revolving doors** | 3 | 0 | 0 | 0 | 72 | 7 | 0 | 15 |
| **Crowded places** | 3 | 0 | 0 | 0 | 72 | 3 | 0 | 5 |
| **Sudden change** | 3 | 0 | 0 | 0 | 72 | 9 | 1 | 49 |
| **Obstacle** | 3 | 0 | 0 | 0 | 72 | 6 | 0 | 15 |
| **Zebra crossing** | 3 | 0 | 0 | 0 | 72 | 0 | 0 | 0 |
| **Dual-tasking** | 6 | 0 | 0 | 0 | 72 | 7 | 0 | 25 |
| **Escalator** | 6 | 0 | 0 | 0 | 72 | 7 | 0 | 21 |
| **Time stress** | 6 | 1 | 0 | 23 | 72 | 0 | 0 | 0 |
